# Effect of Water Uptake, Adhesion and Anti-Corrosion Performance for Silicone-Epoxy Coatings Treated with GLYMO on 2024 Al-Alloy

**DOI:** 10.3390/polym14153076

**Published:** 2022-07-29

**Authors:** Xin Yuan, Yilin Du, Zhihai Lin, Zhiqiang Liu, Lin Gu

**Affiliations:** 1 School of Chemical Engineering and Technology, Sun Yat-sen University, Zhuhai 510275, China; duyilin@foxmail.com (Y.D.); linzhihai@foxmail.com (Z.L.); 2 Advanced Material Test Center, School of Mechanics and Civil&Architecture, Northwestern Polytechnical University, Xi’an 710129, China; liuzhiqiang@mail.nwpu.edu.cn

**Keywords:** silicone-epoxy coatings, 3-glycidoxypropyltrimethoxy silane, EIS, DSC, water uptake, model validation, Al-alloy

## Abstract

Water uptake, adhesion and corrosion performance of silicone-epoxy coating on 2024 Al-alloy treated with different GLYMO were systematically studied by gravimetry, electrochemical measurements, DSC, pull-off adhesion and salt spray tests. The results showed that GLYMO not only enhanced the cross-linking of the silicon-epoxy coating but also enhanced the bonding between the coating and the Al-alloy interface. This gives the coating better wet adhesion, less water absorption and improves the corrosion resistance of the coating. The micro-nano silane layer, preferentially between the coating and Al-alloy oxide layer, was validated by the model of the water concentration jump.

## 1. Introduction

Organic coatings have the advantages of solid chemical resistance and strong adhesion with metal surfaces and have been widely used in metal corrosion protection [1,2,3]. One of the main challenges of using organic coatings is the absorption and penetration of water and corrosive ions. Once the corrosion ions reach the coating/metal interface, the metal substrate will be corroded, leading to delamination between the coating and the protected metal and degrading the coating [4,5,6,7]. Therefore, the barrier performance of the coating to water, oxygen and corrosive ions is related to the protective effect of the coating [8,9,10,11].

One of the most common polymer matrices in the coatings is a silicone-epoxy resin used to protect Al-alloy. Compared with epoxy resin, silicone–epoxy resin has UV resistance, colour retention, excellent mechanical properties, low water permeability, strong adhesion to the metal substrate and high corrosion resistance [4,12,13,14,15,16,17]. However, in an actual service environment, due to the hydrophilic chemical groups in the silicone-epoxy coating structure, such as hydrolysable alkoxy, carboxyl and amino groups, the silicone-epoxy coating will absorb the water and decrease the wet adhesion in the corrosion solution, thus reducing the protective effect on the metal substrates.

Organo-silanes or Organofunctional silanes with the general structure are well-known coupling agents with organic and inorganic properties, which enable them to act as bridging hybrid molecules across the interface of two different surfaces. The bridging structure of organo-silane has important technical significance in surface modification, adhesion promotion and polymer crosslinking [12,13,14,15,16]. When silane coupling agents are used as crosslinking agents, they react with the polymer, causing trialkoxy silane alkyl to be grafted to the backbone of the polymerisation, cross-linked by the siloxane chain, and form a stable three-dimensional structure. When a silane coupling agent is used as an adhesion promoter, it can react with a metal matrix to form a Me-O-Si bond.

**Figure 1:**
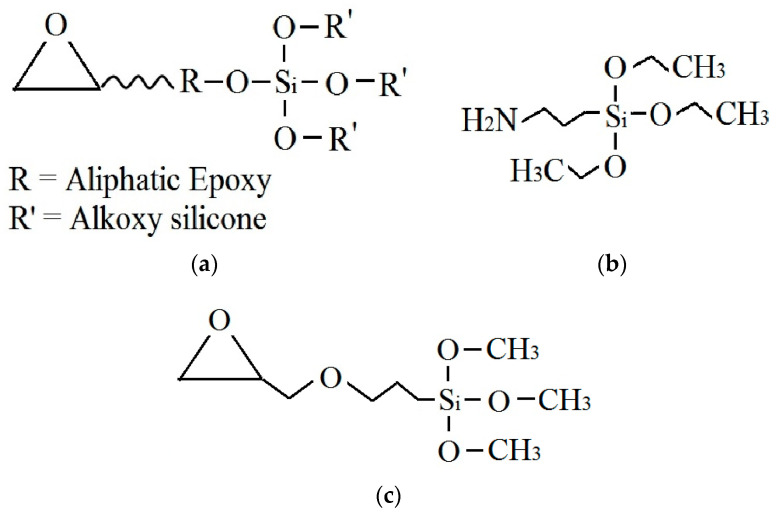
The chemical structure of the main coating components: (**a**) silicone-epoxy resin; (**b**) hardener amino-silane; (**c**) 3-glycidoxypropyltrimethoxy silane.

3-glycidopropyltrimethoxy silane (GLYMO), also known as a silane coupling agent, is a kind of organic silane with three functional groups of an epoxy terminated (as shown in Figure 1). It has been found that GLYMO could improve the adhesion of polymer coating, reduce the coating’s water absorption, and improve the coating system’s corrosion resistance [17,18,19,20]. GLYMO added in the coating has two leading component roles. First, it hydrolyses to form silicon alcohol (Si-OH), which strengthens the polymer resin’s bone structure, increasing the coating’s resistance against electrolyte penetration. Second, adding silane components can also enhance the binding between the coating and the metal matrix by the Si-O-Me covalent bond. Some researchers have shown that various silane monomers can be successfully modified by simple physical doping or chemical grafting, which can significantly improve the corrosion properties of epoxy coatings [21,22,23,24]. This paper will study the water uptake and protective properties of GLYMO-modified silicone-epoxy coatings in this paper.

Electrochemical impedance spectroscopy (EIS) is a well-known electrochemical technique used to evaluate the performance of organic coatings. It has proven to be a powerful tool for obtaining specific parameters of coating/metal systems [14,15,25,26,27,28,29,30]. The coating resistance, capacitance and other parameters obtained from the equivalent circuit (EECs) can quantitatively reflect the corrosion resistance of the coating. It is generally believed that the electrochemical behaviour of the coating/metal in the electrolyte includes transport and diffusion of aggressive ions, electrochemical reactions at the interface, and further deterioration (i.e., mass production of corrosion products and interfacial stratification), leading to complete coating failure [31,32,33,34,35,36].

In this paper, 3-glycidyloxy-propyltrimethoxysilane (GLYMO), a silane monomer, is directly combined with a silicone epoxy/amino coating system at room temperature. The purpose of adding silane monomers to silicone-epoxy/amino is to inhibit the water uptake of the coating, strengthen the combination of metal/coating interface, and improve the protective performance of the coating.

## 2. Experimental Details

### 2.1. Materials

The resin used in this experiment is SILIKOPON@EF silicone-epoxy resin from Evonik industries, the epoxy equivalent of 450 g/mol has excellent corrosion and chemical resistance. The curing agent is selected from Evonik Dynasylan@AMEO, and the hydrogen equivalent is 110 g/mol. The weight ratio of resin and curing agent is 4:1. The silane monomer, 3-glycidoxypropyltrimethoxy silane (GLYMO), is purchased from Dynasylan GLYMO, EVONIK industries, Germany. The chemical structure of the three main coating components is shown in Figure 1. Before use, the resin is diluted to suitable viscosity with n-butanol and butyl acetate. 2024 Al-alloy plates are obtained from Changsha Metal Products Co., Ltd., Changsha, China.

### 2.2. Sample Preparation

2024 Al-alloy was used as the metal matrix of the coating sample, which is a 150 mm × 70 mm × 1 mm rectangular plate. The chemical composition is shown in Table 1. First, the surface of Al-alloy was polished with sandpaper with a 2500 grit finish to remove the thick oxide film and residual oil impurities on the surface of Al-alloy. Then, the sample was placed in acetone solution for ultrasonic cleaning for 30 min. After cleaning, it is then washed with deionised water and dried in a vacuum oven before spraying.

Different amounts of GLYMO were added to the silicone-epoxy/amino-silane system, i.e., the mass ratio (to silicone-epoxy resin) was 0%, 0.75%, 1.5% and 2.25%, respectively. Coating A, B, C and D were applied on Al-alloy by air spraying. After spraying, the sample was placed in a ventilated and dry place for curing for two weeks. The coating thickness was measured by a German QNix 4500 thickness tester. The coating thickness was about 25±1 μm measured by scanning electron microscope and digital thickness gauge QNix 4500.

### 2.3. Gravimetric Experiments

At room temperature, the coating was attached to the metal substrate to measure the water uptake. To minimise the quality difference between the Al-alloy plate and the coating, Coating A, B, C and D (25±1 μm) were coated on the aluminium foil (purity 99.0%) with a thickness of 50 μm. The surface of aluminium foil was not treated, and the coatings were cured at room temperature for 7 days.

Square samples (6 cm × 6 cm) were cut from the coated foils. Each sample was weighed to the nearest 0.1 mg on a Mettler balance before immersion in 200 mL of 5 wt.% NaCl solution. Samples were taken from the NaCl solution regularly, and the excess water on the coating surface was carefully removed with filter paper and then weighed. At the end of the exposure, the coatings were stripped from the foil piece by piece, and the foils were weighed. The sample mass before immersion, after immersion and after coating removal represents $m_{1}$, $m_{2}$ and $m_{0}$, respectively. Each value was obtained by averaging at least 5 measurements.

The mass fraction of water ($\phi_{m}$) absorbed by the coating for each exposure time was calculated as:

(1) $$\phi_{m}=\frac{m_{2}-m_{1}}{m_{2}-m_{0}}\times 100\%$$

### 2.4. Electrochemical Measurements

**Figure 2:**
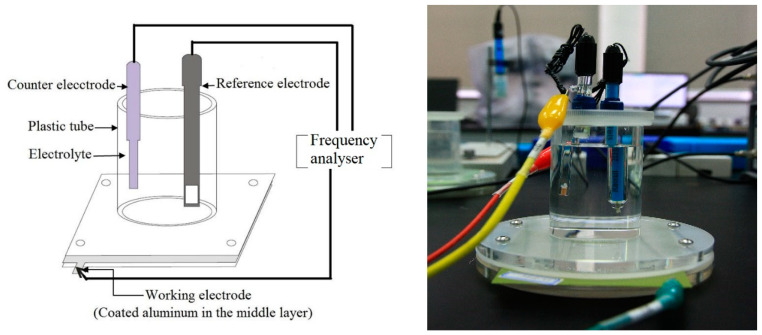
Three-electrode system used for the immersion test and EIS measurements on the coated Al-alloy.

A classical three-electrode system was used for electrochemical testing, in which the electrolyte solution was 5 wt.% NaCl solution. Figure 2 shows the schematic diagram of the immersion test and EIS measurement. The coating/metal system was used as the working electrode, the platinum electrode as the auxiliary electrode and the saturated calomel electrode (SCE) as the reference electrode. The whole electrochemical device was placed in a Faraday cage (shielding interference) for testing. The experimental instruments used in the electrochemical tests were Wuhan Coster CS350 electrochemical Workstation and the Corr-Test electrochemical test system. A sinusoidal potential with an amplitude of 20 mV was applied to the system to disturb it. The frequency scanning range was 10^−2^~10^5^ Hz. The electrolyte solution was changed weekly. Z-View 2 software was used to process and analyse the experimental data.

Considering the system’s non-ideal capacitance characteristics, a constant phase element (CPE) is used to replace all capacitors in EECs to obtain a more accurate fitting result. The impedance of CPE is defined as follows [10,25,26,27,28,29]:

(2) $$Z(j\omega )={\left(Y_{0}\right)}^{-1}{\left(j\omega \right)}^{-n}$$

where $Y_{0}$ is the CPE-constant with the units of ${\mathrm{Fcm}}^{-2}s^{(n-1)}$, j the imaginary number, *n* the CPE-power (0≤n≤1), and ω is the angular frequency (ω=2πf, f is the frequency). The CPE is a pure capacitance for *n* = 1, in which the derivation of *n* from the unit is due to the dispersion effect.

Scanning electrochemical microscopy (SCEM) is an effective in situ method for studying coated or uncoated metals [30,31,32,33,34,35,36]. The SCEM test was performed in the VersaScan scanning electrochemical microscope (AMETEK advanced measurement technology instrument in the United States). Ag/AgCl (saturated KCl) was used as the reference electrode, the Pt wire was used as the counter electrode, and the 15 μm Pt microelectrode was used as the working electrode. There are two measurement modes. The feedback method is used to monitor the swelling behaviour of the complete coating. The other is the redox competition method to measure the oxygen concentration in the scratch defect area.

The complete coating was approached to the surface in 0.5 mm ferrocenyl alcohol + 0.1 mL NaCl solution. The microelectrode is located about 25 microns above the coating. The tip current is measured immediately at +0.8 V vs. Ag/AgCl in the same solution to obtain the diffusion-limited tip current curve at selected locations on the coating for coating swelling measurement. The needle tip was scanned at the centre line of the 550 μm artificial holes, and the feedback current of the defect sample was recorded.

### 2.5. Differential Scanning Calorimeter (DSC)

The glass transition temperature ($T_{g}$) of silicone-epoxy hybrid coating before and after impregnation was measured by differential scanning calorimetry (DSC). The measurement was carried out on the Mettler Toledo dsc1 thermal analysis system. The scanning speed was 15 °C/min, the temperature range was −50~300 °C, and the accuracy was 0.1 °C. The process was carried out according to ASTM/D 3418-82 standard method. $T_{g}$ is half the height of heat capacity change (in the middle of transition). The samples analysed by DSC are made by scraping the coating on 2024 Al-alloys substrate after immersion at different times.

### 2.6. Pull-Off Adhesion and Salt Spray Tests

The pull-out test determined the adhesion of Al-alloy coating before and after immersion in 5 wt.% NaCl solution. The spindle is 20 mm in diameter and glued to the coated surface with epoxy adhesive. After the adhesive is fully cured, separate the coated test area from the area around the spindle by cutting and loading the spindle using A tensile test device (PosiTest at-A, DeFelsko Corporation, Ogdensburg, NY, USA).

A neutral salt spray test was carried out in a salt spray chamber to evaluate the corrosion resistance of the coating samples. According to the ASTM D2803-2015 standard. Cut the coating with a blade to the metal base, sealing the edges and back. The experiment was carried out at 35 °C with 5 wt.% NaCl solution of pH 7 as a corrosive medium. These panels are placed at 45° in a salt spray chamber. Three duplicate samples were tested.

## 3. Results

### 3.1. Water Uptake of the Coatings

**Figure 3:**
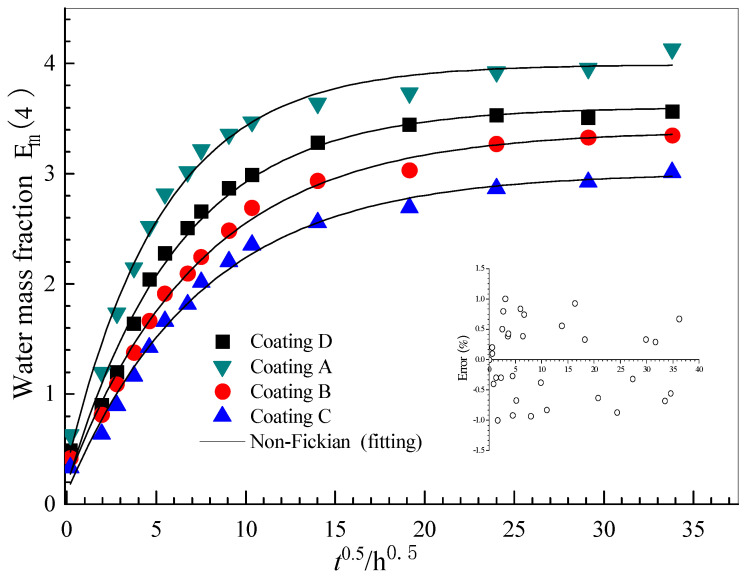
A comparison of the water uptake for the coatings aged in a 5 wt.% NaCl solution obtained via gravimetry.

**Figure 4:**
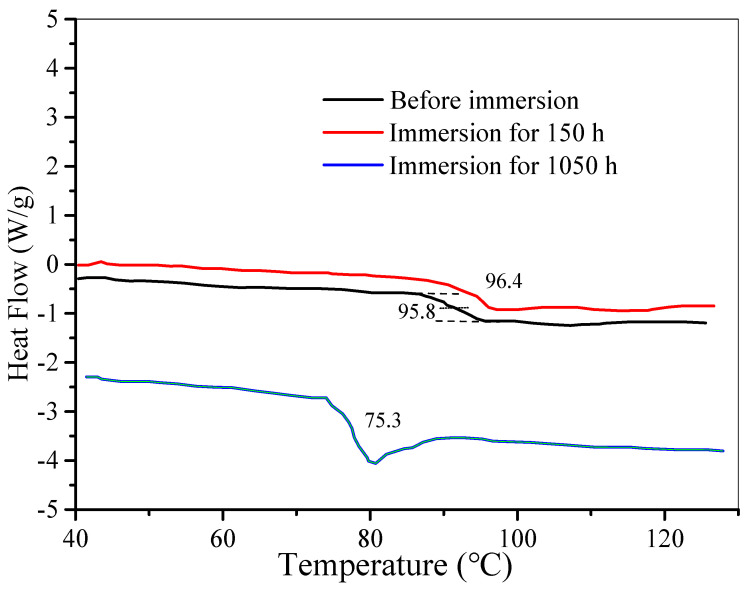
A DSC thermogram of Coating C before and after immersion tests.

Figure 3 shows typical water transport and diffusion of Al-alloy surface coating in 5 wt.% NaCl solution at room temperature. It can be seen that the variation trend of the water mass fraction in the coating is the same. Still, there is no obvious saturation platform after the initial water mass fraction increases. This shows that the ideal Fick-diffusion theory is not applicable. The water absorption curve of the coating shows two steps of water transfer and diffusion: the first Fick-diffusion step and the second non-Fick diffusion step. Nguyen Dang et al. [18] previously clarified the relationship between water mass fraction and the epoxy system’s square root of time evolution. According to the method proposed by the author to determine the parameters of water transport and diffusion, a modified non-Fick relation is used [7,9,10,12,18]:

(3) $$\phi_{t}=\phi_{s}\left(1-\frac{8}{\pi^{2}}\sum_{n=0}^{\infty }\frac{1}{{\left(2n+1\right)}^{2}}\exp\left[\frac{-{\left(2n+1\right)}^{2}D\pi^{2}}{4l^{2}}t\right]\right)+\frac{S_{c}}{l}\sqrt{t}$$

where, $\phi_{t}$ is the water absorption at time *t*, $\phi_{s}$ is the water absorption at saturation, *l* is the coating thickness, and $S_{c}$ is the swelling coefficient from mass measurements ($\mathrm{kg}\cdot \mathrm{cm}\cdot s^{-0.5}$). The NLLS fitting program developed under the Mat Lab environment is used to fit the EIS experimental data with Equation (3), and satisfactory results (relative error < 1.5%). The fitting curve of the coating/Al-alloy system is shown in Figure 4. Table 2 lists the water absorption characteristics of all samples derived from NLLS fitting and calculation results.

The results show that the water diffusion coefficient and saturated mass fraction of the incorporated coatings (i.e., Coatings B, C and D) are lower than that of Coating A, thus reducing the water permeability in the combined coatings. In addition, in the high content coating, the existence of GLYMO has a more significant delay effect on water permeability. This indicated that the water resistance of the coating is improved by adding GLYMO into the silicone-epoxy coating. However, for Coating D, the water diffusion coefficients and saturated mass fractions are higher than that of Coating C. The reason for this may be that the excessive hydrophilicity of unreacted hydrolysed alkoxy (-OCH3) promotes water permeation; this is discussed below.

### 3.2. T_g_ Variation after Water Permeation

The $T_{g}$ change before and after immersion reflects the degree of plasticisation of the polymer matrix and the interaction between water and resin. It is an essential parameter of the coating and is closely related to the density and crosslinking degree of the coating [9,10,16,25,26]: a higher crosslinking degree will cause a higher value of $T_{g}$. After impregnation, the $T_{g}$ value of the silicone-epoxy coating usually decreases because the absorbed water molecules may break the hydrogen bonds between the chains.

From DSC experiments (Figure 4), the $T_{g}$ values for Coatings C before and after immersion in aqueous NaCl solution were obtained. Table 3 lists the $T_{g}$ values of Coatings A, B, C, and D. As mentioned above, $T_{g}$ in Coating A is usually reduced after water penetration. However, $T_{g}$ of coating B, C and D increased slightly after soaking for 150 h. The increased value indicates the crosslinking degree of GLYMO-incorporated coatings is improved after immersion. After 1050 h, the decreased values for Coating B, C and D (shown in Table 3) indicate that the polymer has undergone a certain degree of degradation under the action of permeating water. These suggest that in GLYMO-incorporated coatings, the absorbed water has two competitive effects on the coating: one is the favourable effect of the reaction between water and silane components; the other is the adverse effect on the coating, as mentioned before, resulting in the reduction of $T_{g}$. The reaction between water and silane components is due to the cured coating still containing hydrolysed alkoxy (-OCH_3_), which is hydrolysed to form silanol (Si-OH) under the action of water. Silanol can be condensed to form a Si-O-Si structure. Therefore, the hydrolysis of the alkoxy group consumes the absorbed water, resulting in a decrease in water permeability. On the other hand, the formation of the Si-O-Si structure will increase the degree of cross-linking, thereby increasing and decreasing water permeability. Indeed, formation of the Si-O-Si bond can be used as a repair agent for polymer coating when GLYMO-incorporated coating is used in a humid environment.

Table 3 also shows that with the increase of GLYMO content, $T_{g}$ of the coating increases firstly and then decreases. The maximum value (~95.8 °C) of Coating C is in good agreement with the coating’s current density and water permeability.

Furthermore, it is necessary to study the influence of GLYMO content on the defensive performance of silicone-epoxy coatings.

### 3.3. Anti-Corrosion Performance of Silicone-Epoxy Coatings with GLYMO

**Figure 5:**
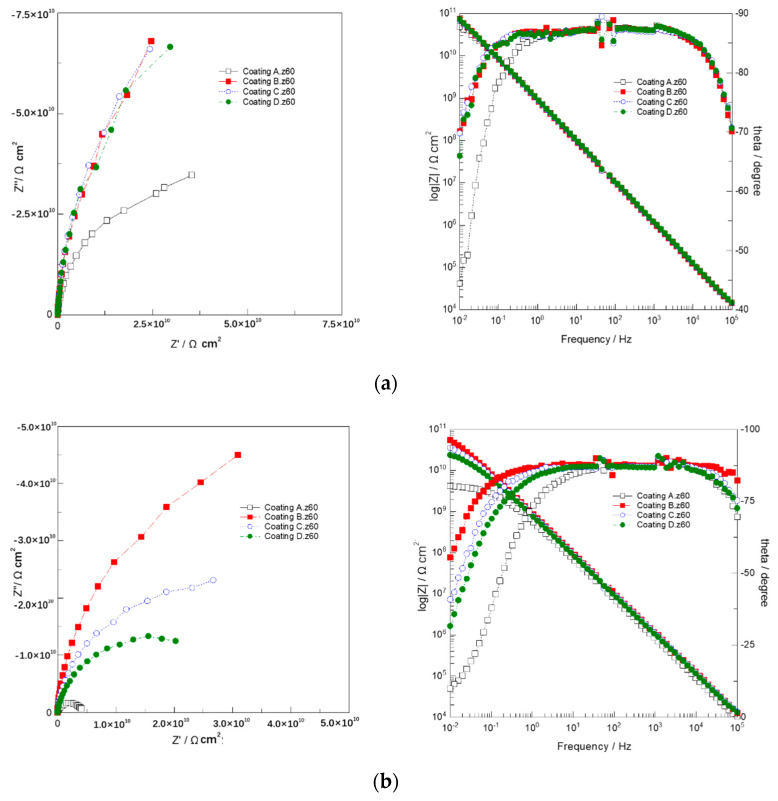

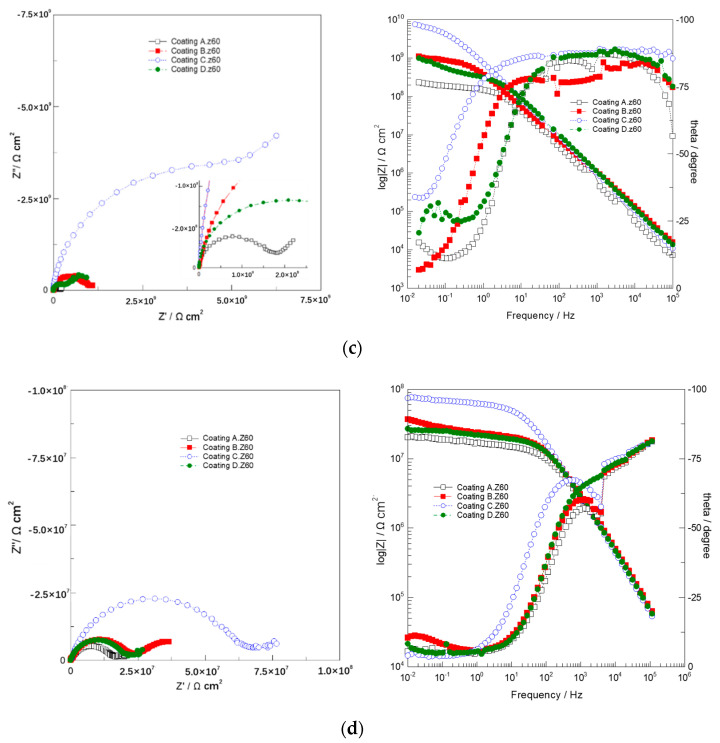
Nyquist and Bode diagrams of EIS measurements of 2024 Al-alloy coated with the coatings containing different ratios of GLYMO immersed in 5 wt.% NaCl solution. (**a**) 0.5 h; (**b**) 150 h; (**c**) 690 h; (**d**) 1050 h.

**Figure 6:**
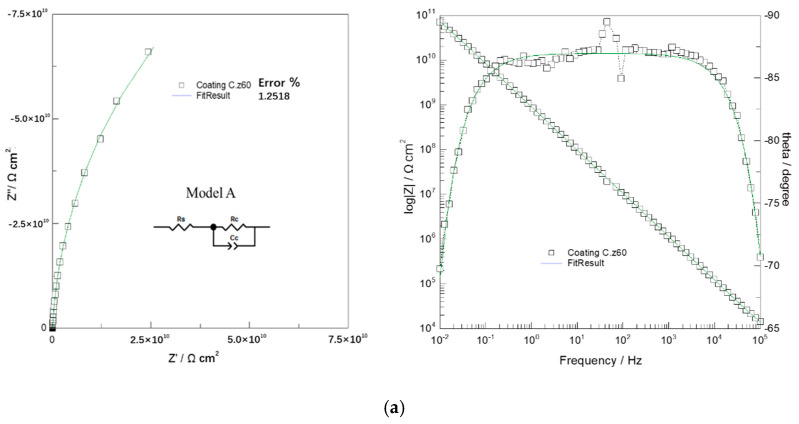

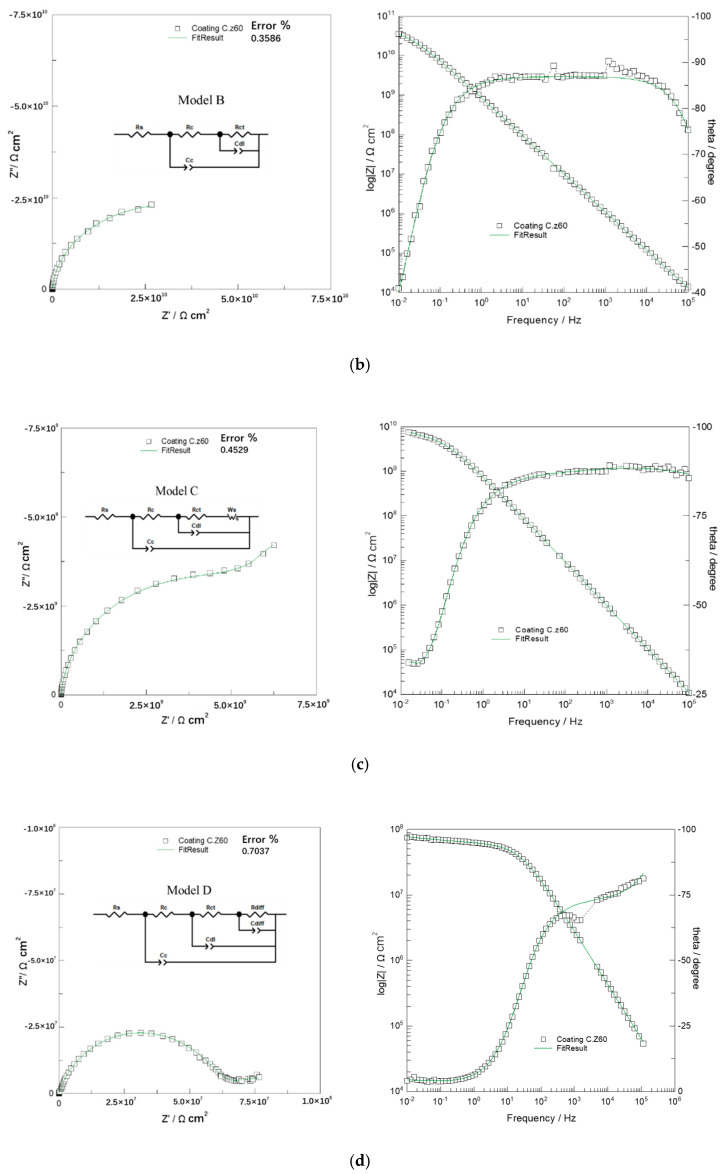
An example of the impedance data of Coating C and fitted curves with the indicated EEC models. (**a**) 0.5 h; (**b**) 150 h; (**c**) 690 h; (**d**) 1050 h.

Figure 5 shows the Nyquist diagram and Bode diagram of EIS data of 2024 Al-alloy coated with coatings A, B, C, and D s under different immersion times. Taking the Coating C/Al- alloy system as an example, the impedance response was analysed using four typical EECs (inserted in Figure 6). Figure 6 shows the fitting curves of different models at different times.

During the initial immersion stage (0.5 h), only four arcs with high impedance values were observed in Nyquist plots, i.e., typical capacitance behaviour and barrier coating characteristics. Bode also shows four lines corresponding to the size of the complete coating. Model A, shown in Figure 6a, describes the impedance behaviour of the coating system during initial immersion. $R_{s}$ corresponds to the uncompensated resistance between the reference electrode and the working electrode. $C_{c}$ is coating capacitance. $R_{c}$ is called pore resistance (i.e., coating resistance), which is actually due to the formation of ionic conductive paths in the coating after the ions, oxygen and water.

After immersion for 150 h, four capacitance loops are observed in the Nyquist diagram in Figure 5b, where impedance semicircles in the high-frequency range correspond to coating properties, and incomplete impedance semicircles in the low-frequency range correspond to electrochemical reactions at coating/metal interfaces. It is considered that water and oxygen molecules reach the surface of the Al-alloy and that electrochemical reactions may occur at the metal/coating interface. Model B is introduced to describe the impedance response at this time. $C_{\mathrm{dl}}$ represents the double-layer capacitance and $R_{\mathrm{ct}}$ represents the corresponding charge transfer resistance (i.e., polarisation resistance). Compared with Coating A, there is not much difference between Coatings B, C and D, which shows that adding GLYMO to epoxy-silicone coating can improve the corrosion resistance of the coating.

After immersion for 650 h, it can be seen from Figure 5c that there are four semicircular circles at the high frequency, and the four lines form a certain angle with the real axis at the middle and low frequencies. Dynamics change from charge transfer control at high frequency to diffusion control at low and medium frequency, and the influence of the finite diffusion layer is dominant. Model C (Figure 6c) was introduced to fit the impedance response of the coating systems, in which the diffusion of erosive ions and corrosion products at the coating/substrate interface was represented by Warburg impedance $W_{s}$. In the Bode diagram, ${\left|Z\right|}_{0.01}$, the impedance modulus of 0.01 Hz can be used to evaluate the protective performance of the coating quickly. As seen from the Bode diagram of Figure 6c, the impedance modulus of coating C is much higher than that of the other three coatings. It can be inferred that with the increase of GLYMO content, the corrosion resistance of the silicone-epoxy coating can be improved, and there is an appropriate value (~1.5%), coating C.

After 1050 h of immersion, the Nyquist diagram changed significantly again (as shown in Figure 5d), and a large tail appeared in the low-frequency region. These tails may be related to the infinite layer diffusion process caused by corrosion products on the surface of the active electrochemical site. The introduced Model D (i.e., Figure 6d) contains the diffusion impedance, which includes the diffusion capacitance ($C_{diff}$) and the diffusion resistance ($R_{diff}$). This diffusion behaviour is not the ideal Warburg impedance, resulting in dispersion coefficient *n* deviating from 0.5 [26,27,28,29,30].

From the above analysis, it can be known that the smaller semicircle diameter may be related to more ion conduction in the coating, because the long-term immersion makes the electrolyte penetrate more into the pores in the coating, forming a perforation, and then reaches the underlying Al-alloy substrate. The semicircle diameter of Coating C is more significant than that of the other three coatinhigh-frequency frequency ranges, indicating that 1.5 wt.% GLYMO can improve the pore resistance of the silicon-epoxy coating. As seen in Figure 5d, silicone-epoxy coatings’ anti-corrosion performance of silicone-epoxy coatings with GLYMO is better than that without GLYMO. The corrosion results in NaCl solution also show that the pore resistance of silicone-epoxy coating increased with the increase of GLYMO content because the hydrolysis and condensation of GLYMO in the matrix reduced the through-hole ratio, with the appropriate value (~1.5%) of Coating C. The impedance modulus (unit: $\Omega \cdot {\mathrm{cm}}^{2}$ ) of Coating C at the early and late immersion stages are respectively $7.03\times 10^{10}$ and $7.47\times 10^{7}$. Compared with other similar silane-modified epoxy coatings [28,29], Coating C also has better protective performance.

**Figure 7:**
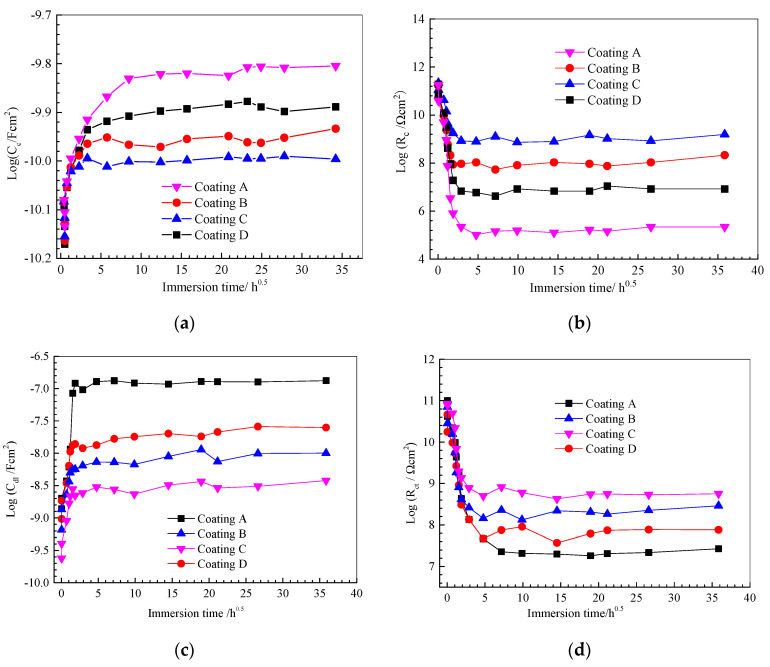
Evolution of the electrical parameters $C_{c}$ (**a**), $R_{c}$ (**b**), $C_{\mathrm{dl}}$ (**c**) and $R_{\mathrm{ct}}$ (**d**) derived from EIS data for the coatings during the immersion in 5 wt.% NaCl solution.

Figure 7 shows the evolution of four typical electrical parameters ($C_{c}$, $R_{c}$, $C_{\mathrm{dl}}$ and $R_{\mathrm{ct}}$). The coating resistance $C_{c}$ is believed to be related to the water blocking performance of the organic coating, and it increases due to water absorption during the immersion [10,11,12,13,14,15,16,17,18]. As a primary trend, the $C_{c}$ value increases rapidly at the initial immersion stage, and then stabilises in all coating systems (i.e., Figure 7a), regardless of GLYMO. The results show that the $C_{c}$ values of the GLYMO-incorporated coatings are lower than that of the pure epoxy coating, indicating that the coating’s water absorption and barrier properties are improved. Similar trends can also be found for the water mass fraction of the coatings (i.e., Figure 3), and the $R_{c}$ (i.e., Figure 7b) with the opposed direction to $C_{c}$. Higher $R_{c}$ and lower $C_{c}$ indicate that the doping of GLYMO can improve the resistance to ion and water permeability.

$C_{\mathrm{dl}}$ and $R_{\mathrm{ct}}$ are two critical parameters directly related to the protective performance of coating samples. They are used to measure the total number of active centres in the electrochemical corrosion reaction of the metal/electrolyte interface. The change of ($C_{\mathrm{dl}}$$R_{\mathrm{ct}}$) is similar to that of ($C_{c}$$R_{c}$), which reflects the development of the double-layer structure of the metal/electrolyte interface and the progress of corrosion reaction (i.e., Figure 7c,d). In addition, compared with the pure silicone-epoxy coating (i.e., Figure 7c,d), the $C_{\mathrm{dl}}$ values of GLYMO-incorporated coatings are lower and higher, indicating that the addition of GLYMO can effectively inhibit the corrosion reaction of the coated Al-alloy. The reason for this result can be explained as hydrolysed free GLYMO silane molecules in the coating form hydrophilic silanol functional groups, which can react with -OH on the surface of the substrate. When the precursor solution acts on the surface of the substrate, the above reaction consumes the silane near the interface between the substrate and the solution. The resulting concentration gradient drives silane diffusion to the substrate surface, forming a silane-rich interface layer [30]. Later discussions will verify whether silane layers exist.

### 3.4. Wet Adhesion and Salt Spray

Besides the better barrier property, the coating with GLYMO has better adhesion to Al-alloy. Table 4 summarises the results of the pull-off adhesion measurements. The results show that the wet adhesion of silicone-epoxy in NaCl solution decreases significantly, while that of GLYMO-incorporated coatings decreases slightly. Compared with silicone-epoxy coating, the excellent adhesion between GLYMO-incorporated coatings and Al-alloy substrate is achieved by enriching silane on the substrate [30]. The resulting silane interlayer repels water and salt, inhibiting degradation and adhesion loss at the substrate/coating interface.

**Figure 8:**
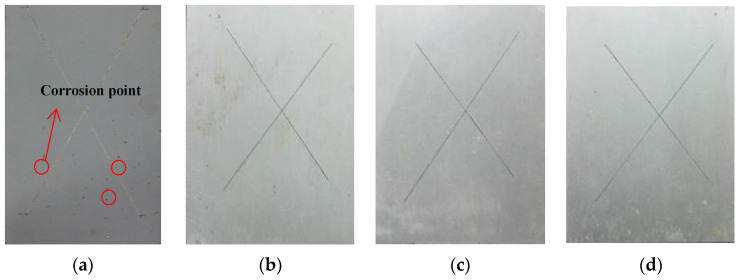
Salt spray results for 2024 Al-alloy samples coated Coating A (**a**); Coating B (**b**); Coating C (**c**); Coating D (**d**) after 1000 h.

Furthermore, the corrosion resistance of the coatings was evaluated by salt spray tests. Figure 8 shows the results for the coated samples after 1000 h of exposure. The surface of silicone-epoxy coating has a little bit of corrosion area. In addition, it is layered near the scribe, indicating poor adhesion with the substrate. GLYMO-incorporated coatings have almost no corrosion area.

### 3.5. SCEM

**Figure 9:**
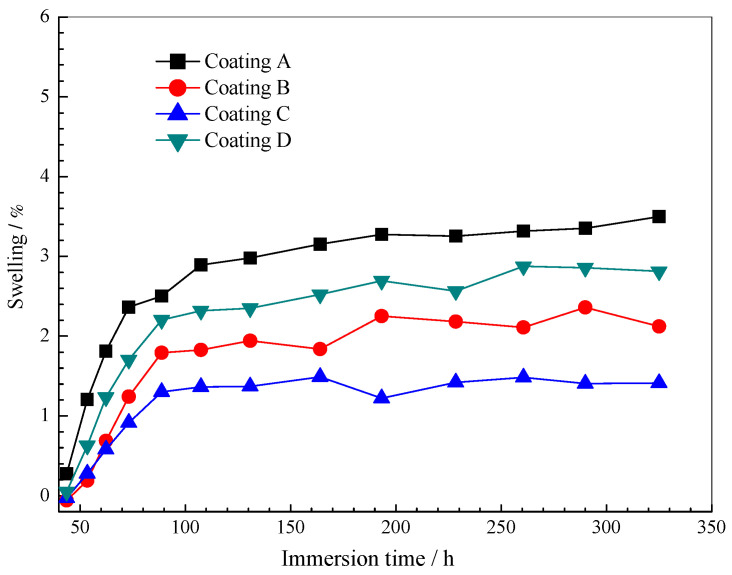
The evolution of coating swelling during exposure to 5 wt.% NaCl solution.

Here, with the help of the approach curve, the tip-substrate distance can be derived from the reduced feedback current [31,32]. The SCEM feedback mode was used to monitor the swelling of the silicone-epoxy coating by reducing the tip to substrate distance obtained by the feedback current. As shown in Figure 9, the four coatings swell significantly at the beginning of the immersion. After that, no significant swelling is observed for these coatings. After immersion for 100 h, the final swelling rate of silicone-epoxy coating containing GLYMO is less than that of coating without GLYMO. This is the same as the swelling coefficient $S_{c}$ (i.e., Table 2) and shows that the bulk structure of the silicone-epoxy coating is enhanced by adding a GLYMO silane agent.

**Figure 10:**
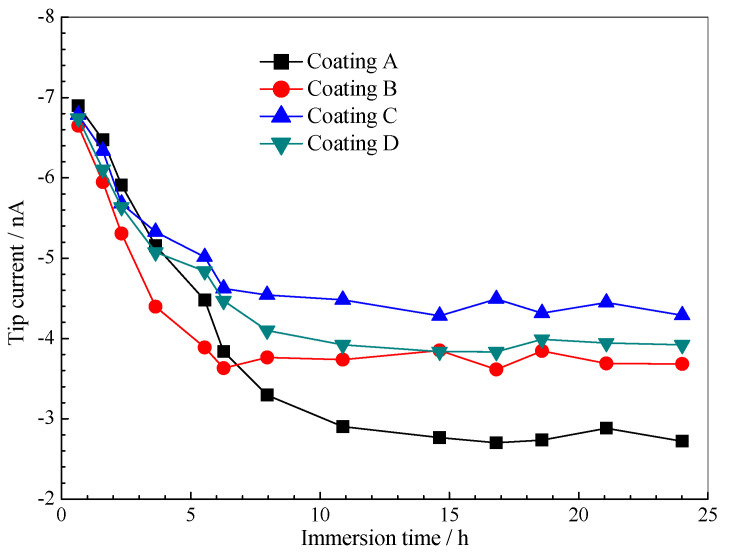
The evolution of tip current for O_2_ reduction over the scratch in 5 wt.% NaCl solution.

In the immersion process, the active dissolution of Al-alloy will expend the dissolved oxygen in the solution near the scratch. The oxygen concentration change can be detected at negative potential platinum microelectrode [33,34,35]. Therefore, the local corrosion behaviour of the Al-alloy matrix under scratches was monitored using SCEM oxidation-reduction competition mode. Figure 10 shows the change of tip current with immersion time on the scratch. In the early stage of immersion (11 h), the tip current of the silicone-epoxy coating dropped sharply, which indicated that the active dissolution process of the Al-alloy at the scratch is speedy. After that, the current remains unchanged because the corrosion reaction is stable. The needle current decreases slowly for the three kinds of GLYMO-doped coatings, but the plateau current is about 1–1.5 times that of pure coatings. This result indicates that the GLYMO silane component has an inhibitory effect on the silicone-epoxy coating.

## 4. Discussion

The results of electrochemical measurement and salt spray tests showed that adding GLYMO can significantly improve the corrosion resistance of the silicone-epoxy coating. The structural enhancement can explain this phenomenon as a result of GLYMO incorporation. On the one hand, GLYMO increases the crosslinking density of the polymer resin, thus enhancing the coatings’ bulk structure. On the other hand, the preferential formation of the micro-nano silane layer between the silicone-epoxy coating and Al-alloy leads to the enhancement of the interface structure [35]. However, whether there is a silane layer at the interface should be discussed to enhance the interface bonding.

**Figure 11:**
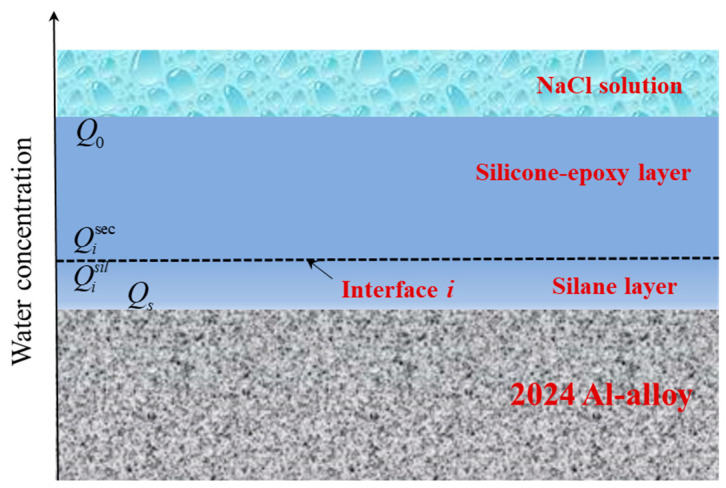
A schematic illustration of the water concentration jump on the silane layer/silicone-epoxy interface.

It is known from our previous studies [36] that if there is a silane film at the interface, it will lead to the water concentration non-continuity at the interface, that is, there is a water concentration jump. Therefore, the water concentration jump model was used to verify whether GLYMO preferentially forms silane film at the coating interface. As shown in Figure 11, interface *i* represents the boundary between the inner silane layer and the outer silicone epoxy layer. $Q_{0}$ and $Q_{i}^{\sec}$ are the water absorption concentrations of the surface and boundary of the silicone epoxy resin layer respectively. $Q_{i}^{sil}$ represents the surface water absorption concentration of the silane layer. $Q_{s}$ is the concentration of absorbed water close to Al-alloy. When Fick’s law describes water diffusion, the amount of water absorbed per second into the silicone-epoxy and silane layers as immersion time increases are expressed by Equations (4)–(6) as following [36]:

(4) $$j_{\sec}=\frac{D_{\sec}(Q_{0}-Q_{i}^{\sec})}{L_{\sec}}$$

(5) $$j_{sil}=\frac{D_{sil}(Q_{i}^{sil}-Q_{s})}{L_{sil}}$$

(6) $$j_{\mathrm{com}}=\frac{D_{com}^{M}(Q_{0}-Q_{s})}{L_{com}}$$

where $j_{\sec}$, $j_{\mathrm{sil}}$ and $j_{com}$ are the absorbed water flux per unit area per second in the outer silicone-epoxy layer, the inner silane layer and the coating with GLYMO; $D_{com}^{M}$ is the effective coefficient of water diffusion for the composite coating calculated by the model validation; $L_{\sec}$, $L_{sil}$ and $L_{com}$ are the thicknesses of the outer silicone-epoxy layer, inner silane film and the coating with GLYMO. A coefficient *k* is introduced to characterise the water concentration jump on the silicone-epoxy/silane film boundary and defined by Equation (7) as follows:

(7) $$k=\frac{Q_{i}^{\mathrm{sil}}}{Q_{i}^{\sec}}$$

Considering that the coating with GLYMO exhibits excellent barrier properties after a prolonged immersion time and the diffusion process in the coating with GLYMO attains a relatively steady state, the concentration $Q_{s}$ of the absorbed water close to the Al-alloy is insignificant, $Q_{s}\approx 0$, $j_{\sec}\approx j_{\mathrm{sil}}\approx j_{com}$. It can be obtained as follows:

(8) $$\frac{D_{com}^{M}}{L_{com}}=\frac{kD_{sil}D_{\sec}}{D_{\sec}L_{sil}+kD_{sil}L_{\sec}}$$

In this study, $L_{com}\approx L_{\sec}+L_{sil}$, $L_{\sec}\approx 25$ μm and $L_{sil}\ll L_{\sec}$, then Equation (9) can be obtained as follows:

(9) $$D_{com}^{M}=\frac{\left(25+L_{sil}\right)kD_{sil}D_{\sec}}{D_{\sec}L_{sil}+25kD_{sil}}\approx \frac{D_{sil}D_{\sec}}{D_{\sec}/25k+D_{sil}}$$

It is considered a limiting case for *k* (k « 1, i.e., $Q_{i}^{sil}$ « $Q_{i}^{\sec}$) as shown in Equation (9), $D_{com}^{M}$ of the coating with GLYMO is discussed: $D_{com}^{M}$ decreases effectively as $D_{\sec}/25k+D_{sil}$ becomes larger, being attributed to the structure of the composite coating. This indicates that a suitable barrier property of the inner silane film increases the water diffusion resistance of the composite coating. Therefore, the $D_{com}^{M}$ value will be calculated. It can be known that $V_{s}^{sil}$ is the volume fraction of water in silane film at saturation, the value of 1.15% [36]; $V_{s}^{\sec}$ is the volume fraction of water absorbed in the silicone-epoxy layer at saturation, the discount of 3.47%. Thus, Equation (10) can be obtained as follows:

(10) $$k=\frac{Q_{i}^{\mathrm{sil}}}{Q_{i}^{\sec}}\approx \frac{V_{s}^{\mathrm{sil}}}{V_{s}^{\sec}}=\frac{1.15}{3.47}=0.33$$

From Equations (9) and (10), $D_{com}^{M}$ can be obtained as follows:

(11) $$D_{com}^{M}=\frac{D_{sil}D_{\sec}}{D_{\sec}/25k+D_{sil}}=\frac{0.95\times 10^{-10}\times 7.72\times 10^{-11}}{0.95\times 10^{-10}/\left(25\times 0.33\right)+7.72\times 10^{-11}}=0.38\times 10^{-10}$$

It can be seen that the $D_{com}^{M}$ value ($0.38\times 10^{-10}$cm^2^ s^−1^) calculated by the validation model considering the water concentration jump, is within the range of the $D_{\mathrm{com}}$ values ($0.35∼0.55\times 10^{-10}$cm^2^ s^−1^) measured by the Gravimetric method. The excellent agreement between the measured and calculated values of water diffusion coefficients confirms the assumption of the water concentration jump at the interface.

## 5. Conclusions

The conclusions drawn from the results of this work are as follows:

(1) Water uptake of silicone-epoxy coatings decreased after incorporated with GLYMO monomer. With the increase of GLYMO content, water of the coatings went through a minimum, corresponding to 1.5% of GLYMO monomer. EIS experiments also show that mixing a certain amount of GLYMO can improve the protective performance of silicone-epoxy coating.
(2) For GLYMO-incorporated coatings, *T*_g_ increased slightly after water permeation because of the self-repairing effect, and finally decreased lower than that of the coating without GLYMO.
(3) SECM measurement showed that the corrosion rate of Al-alloy substrate beneath the artificial defects of GLYMO-incorporated silicone-epoxy coatings was also significantly reduced.
(4) Salt spray and adhesion tests showed the enhancement of interface structure. Adding GLYMO could preferentially form a micro-nano silane layer between the silicone-epoxy coating and Al-alloy. This was validated by the model of the water concentration jump.

## Figures and Tables

**Table 1:** A nominal composition (wt.%) of the 2024 Al-alloy.

Cu	Mg	Mn	Fe	Si	Zn	Ni	Ti	Al
3.8	1.5	0.6	0.5	0.5	0.3	0.1	0.15	balance

**Table 2:** Calculated and fitted water uptake results for the coatings aged in 5 wt.% NaCl solution.

Coatings	*D*/ × 10^−10^ cm^2^·s^−1^	*S*_c_/ × 10^9^ kg·cm·s^−0.5^	100 *V*_s_/%
Coating A	0.95	1.43	3.47
Coating B	0.55	1.17	2.69
Coating C	0.35	1.04	2.35
Coating D	0.47	1.26	2.99

**Table 3:** The *T*_g_ values of the coatings for samples before and after immersion.

Sample	*T*_g_/°C			∆*T*_g_/°C
	Before Immersion	Immersion for 150 h	Immersion for 1050 h	
Coating A	92.8	87.4	65.3	−27.5
Coating B	93.2	95.1	70.9	−22.3
Coating C	95.8	96.4	75.3	−20.5
Coating D	93.6	94.3	69.8	−23.8

**Table 4:** The effects of GLYMO on adhesion strength of the silicone-epoxy coatings.

Sample	Average Adhesion Strength (MPa)				Fracture Mode
	Before Immersion	Coefficient of Variation, CV	After Immersion	Coefficient of Variation, CV	
Coating A	2.55	0.082	1.78	0.103	100% C/M
Coating B	3.15	0.103	2.68	0.087	
Coating C	3.45	0.107	3.12	0.096	
Coating D	3.32	0.095	2.85	0.099	

Note: C/M represents the interface between the coating and metal.
